# Associations between social support, mental wellbeing, self-efficacy and technology use in first-time antenatal women: data from the BaBBLeS cohort study

**DOI:** 10.1186/s12884-018-2049-x

**Published:** 2018-11-12

**Authors:** Samuel Ginja, Jane Coad, Elizabeth Bailey, Sally Kendall, Trudy Goodenough, Samantha Nightingale, Jane Smiddy, Crispin Day, Toity Deave, Raghu Lingam

**Affiliations:** 1 School of Psychology, Ulster University, Cromore Road, Coleraine, Co., Londonderry, BT52 1SA UK; 2 Centre for Innovative Research Across the Life Course (CIRAL), Faculty of Health & Life Sciences, Coventry University, Priory Street, Coventry, CV1 5FB UK; 3 Centre for Health Services Studies, University of Kent, Canterbury, Kent CT2 7NF UK; 4 Centre for Child & Adolescent Health, University of the West of England Bristol, Oakfield House, Oakfield Grove, Clifton, Bristol, BS8 2BN UK; 5 Nursing, Midwifery and Health, Health and Life Sciences, Northumbria University, Coach Lane Campus, Benton, Newcastle upon Tyne, NE7 7XA UK; 6 Department of Psychology, Child & Adolescent Mental Health Service Research Unit, King’s College London, Institute of Psychiatry, Psychology and Neuroscience, De Crespigny Park, London, SE5 8AB UK; 7 Population Child Health Research Group, Women’s and Children’s Health, University of New South Wales, Sydney, Australia

**Keywords:** Antenatal, Pregnancy, Wellbeing, Self-efficacy, Social support, Technology use

## Abstract

**Background:**

Information and communication technologies are used increasingly to facilitate social networks and support women during the perinatal period. This paper presents data on how technology use affects the association between women’s social support and, (i) mental wellbeing and, (ii) self-efficacy in the antenatal period.

**Methods:**

Data were collected as part of an ongoing study - the BaBBLeS study - exploring the effect of a pregnancy and maternity software application (app) on maternal wellbeing and self-efficacy. Between September 2016 and February 2017, we aimed to recruit first-time pregnant women at 12–16 gestation weeks in five maternity sites across England and asked them to complete questionnaires. Outcomes included maternal mental wellbeing (Warwick-Edinburgh Mental Wellbeing Scale), and antenatal self-efficacy (antenatal version of the Tool to Measure Parenting Self-Efficacy). Other variables assessed were perceived social support (Multidimensional Scale of Perceived Social Support), general technology use (adapted from Media and Technology Usage and Attitudes Scale). Potential confounders were age, ethnicity, education, socioeconomic deprivation, employment, relationship status and recruitment site. Linear regression models were developed to analyse the relationship between social support and the outcomes.

**Results:**

Participants (*n* = 492, median age = 28 years) were predominantly white British (64.6%). Half of them had a degree or higher degree (49.3%), most were married/living with a partner (83.6%) and employed (86.2%). Median (LQ-UQ) overall scores were 81.0 (74.0–84.0) for social support (range 12–84), 5.1 (4.7–5.4) for technology use (range 1–6), 54.0 (48.0–60.0) for mental well-being (range 14–70), and 319.0 (295.5–340) for self-efficacy (range 0–360). Social support was significantly associated with antenatal mental well-being adjusting for confounders [adj R^2^ = 0.13, *p* < .001]. The addition of technology use did not alter this model [adj R^2^ = 0.13, *p* < .001]. Social support was also significantly associated with self-efficacy after adjustment [adj R^2^ = 0.14, *p* < .001]; technology had limited impact on this association [adj R^2^ = 0.13, *p* < .001].

**Conclusions:**

Social support is associated with mental well-being and self-efficacy in antenatal first-time mothers. This association was not significantly affected by general technology use as measured in our survey. Future work should investigate whether pregnancy-specific technologies yield greater potential to enhance the perceived social support, wellbeing and self-efficacy of antenatal women.

**Electronic supplementary material:**

The online version of this article (10.1186/s12884-018-2049-x) contains supplementary material, which is available to authorized users.

## Background

Maternal mental health disorders during the antenatal and postnatal periods, most commonly depression and anxiety, are a significant public health problem. In the UK, around 12% of pregnant women experience depression and 13% experience anxiety, with many experiencing both. Rates of depression and anxiety increase to 15 to 20% of women in the first year after childbirth [1]. Worryingly, as many as half of all cases of perinatal depression and anxiety go undetected [2]. Evidence suggests that maternal depression continues to emerge many months after the point where routine depression screening currently occurs [3]. Maternal perinatal mental health disorders are associated with an increased risk of cognitive, emotional and behavioural difficulties in the child, which in turn can lead to adverse long-term life chances [4, 5]. It is estimated that perinatal mental health disorders cost the UK economy £8.1 billion per annum, 72% of these costs being related to adverse impacts on the child [6].

One major factor affecting perinatal mental health is social support. Although definitions of social support vary widely, this concept is often used to refer to the emotional and instrumental assistance received from various sources, with a focus on how the support is perceived by the recipient [7, 8]. A large body of research has demonstrated the link between maternal mental health and social networks, where the most supported mothers experience the best mental health outcomes [9–11]. For example, mothers are five times more likely to experience postpartum depression if they received no support or minimal support after the birth of the baby than women who received appropriate support [12]. Better levels of social support are thought to improve the mother’s ability to cope with stressful life events and to interact with her child in more positive and stimulating ways. Possible reasons for this include easier access to information on developmentally appropriate methods of parenting and the fact that other people can act as buffers against maladaptive parenting and stressful life situations [13]. Despite the increased demands of motherhood, there is some evidence for a decline in social support perceived by new mothers, from pregnancy to postpartum [14]. Stronger social networks during pregnancy can protect against postpartum depression [15], which highlights the role of prevention programmes from an early stage.

With the increasing use of the internet and social media, transformation of the traditional family and austerity cuts to health and social care services [16], there is increasing interest in delivering information and supplement support to pregnant women through information and communication technologies. It has been suggested that the support that perinatal women receive through online technologies can be grouped as emotional support and instrumental support (both formal and informal), as well as community building and protection [17]. The internet is a major source of ante- and postnatal information with as many as three quarters of pregnant women using the internet for medical advice globally [18]. Use of technology has been identified as a way of increasing new mothers’ confidence in mothering by enabling access to information and reassurance about their choices or concerns [19]. It has been reported that online support groups provide women experiencing postpartum depression with a safe place to connect with others and receive ‘information, encouragement and hope’ [20]. Blogging is thought to enhance the connection with extended family and friends, increasing social networks, which in turn has a positive impact on maternal well-being [21]. Another key technology are mobile apps, which should be linked to trustworthy websites, containing short answers to everyday concerns and information on local support services for pregnant and post-birth women [22]. Despite the potential benefits of existing technologies, there is still a lack of quantitative research that assesses the relationship between technology use and social support amongst women in the perinatal period.

The current paper assessed the association between social support and i) mental wellbeing and ii) self-efficacy in a large sample of UK, first-time antenatal women, and how general technology use affected these associations.

## Methods

We used baseline data collected as part of the BaBBLeS study (Bumps and Babies Longitudinal Study), which aimed to assess the impact of a specific mobile software application (app) - Baby Buddy app [23] – on maternal self-efficacy and mental wellbeing at three-month post-partum [24]. In short, this cohort study was conducted in England (UK) to compare both of the above outcomes between first-time prospective mothers who used the Baby Buddy app and women who did not use it, ante- and postnatally, adjusting for key confounders [25]. The current article does not specifically look at the use of the Baby Buddy app but rather investigates the impact of general technology use on the association between social support, maternal mental wellbeing and self-efficacy.

First-time pregnant women aged 16 years or older were recruited in five maternity sites across England between September 2016 and February 2017. To ensure national geographical spread, maternity units were located in different areas of the country: West Midlands (site 1), London (site 2), Lancashire (site 3), East Midlands (site 4) and West Yorkshire (site 5). We aimed to recruit women and collect baseline data during the period of 12–16 weeks gestation.

The maternity services identified eligible women who were then invited to take part in the study in one of two ways. Women were either approached by research midwives when they attended a maternity appointment and were given a recruitment pack, or they were sent a recruitment pack through the post by their local maternity services. The recruitment pack contained information about the study, a consent form, contact sheet, a baseline questionnaire and reply envelope. Women were asked to complete the baseline questionnaire, consent form and contact sheet, and return them in the pre-paid envelope provided. Optionally, the survey (including consent form and contact details) could be completed online. The study questionnaire had been pilot tested and adapted for ease of use and meaning with nine mothers before administration.

The recruitment period lasted 5 months; if a questionnaire was not returned by 2 weeks after the initial questionnaire was sent, women were sent a reminder pack.

Questionnaire data were entered on an online survey platform [26], either directly by participants, or by the research team in the case of participants who had returned paper questionnaires. A 10% subsample of paper questionnaires was randomly chosen and double-data entered to ensure accuracy of data entry. The error rate was less than 2% which was deemed acceptable. This process was carried out in consultation with the local clinical trials unit.

### Ethics

Study ethical approval was gained from NHS Research Ethics Committee (NRES) West Midlands-South Birmingham REC (16/WM/0029) and from the University of the West of England, Bristol Research Ethics Committee (HAS.16.08.001). Research & Development (R&D) departments within each of the study sites confirmed their capability and capacity to conduct the research.

### Measures

The baseline questionnaire is presented in Additional file 1. Questions asked about perceived social support (exposure variable), maternal mental wellbeing and self-efficacy (outcome variables) and sociodemographic data and general technology use (potential confounders). Sociodemographic questions were asked to collect information on age, ethnicity, postcode (from which a socioeconomic deprivation score was derived), relationship status and employment. Recruitment site was determined based on the unique code that identified each questionnaire pack. Participants had the opportunity to write down any comments as free text.

#### Exposure variable

The primary exposure variable assessed in this study was perceived social support, measured using the Multidimensional Scale of Perceived Social Support (MSPSS) [27]. This scale consists of 12 statements about the support received from family (4 items), friends (4 items) and a significant other (4 items), e.g., ‘My family really tries to help me’ or, ‘There is a special person in my life who cares about my feelings’. Participants rated their level of agreement with each statement on a seven-item Likert scale, from 1 (very strongly disagree) to 7 (very strongly agree) (range 1–7). Item scores were summed to provide total scores, both overall (range 12–84) and for each of the three subscales (range 4–28). Higher scores indicated perception of greater social support. The factorial validity and internal reliability of the MSPSS have been demonstrated in a number of studies, with alpha scores from 0.87 to 0.93 [27–29], including amongst pregnant women (alpha 0.92) [30].

#### Outcome variables

The two outcome variables in this study were maternal mental wellbeing and self-efficacy. Maternal mental well-being was assessed by the Warwick-Edinburgh Mental Well-Being Scale (WEMWBS) [31]. This scale comprises of 14 statements describing feelings (e.g., ‘I have been feeling useful’) and functional aspects (e.g., ‘I’ve been dealing with problems well’) over the previous 2 weeks. Items were scored from 1 (none of the time) to 5 (all of the time) and summed to provide an overall score between 14 and 70, where higher scores corresponded to higher levels of wellbeing. This scale has shown good content and criterion-related validity, as well as high test-retest reliability (0.83), in various groups and public health contexts, including parenting programmes [32].

Maternal self-efficacy was measured with a 36-item scale adapted for use in the antenatal period from the tool to measure Parenting Self-Efficacy (TOPSE) [33] to assess parents’ beliefs about their ability to manage their child. This antenatal adaptation was carried out in consultation with the tool developer (SK, one of the co-authors of this paper). All TOPSE statements were reworded to the future tense, e.g., ‘I am able to have fun with my baby’ ➔ ‘I will be able to have fun with my baby’. The scale is divided into six sections, each section containing six items and addressing a different domain of parenting, such as ‘emotion and affection’, or ‘play and enjoyment’. Participants were requested to select how much they agreed with each item, from 0 (completely disagree) to 10 (completely agree) on a Likert scale. Five items were reverse-scored (items 6, 19, 20, 21 and 27). A total sum score was calculated from the 36 items, ranging from 0 to 360, where larger scores indicated greater self-efficacy. The postnatal scale has shown very high internal consistency (alpha 0.96) as well as good content and convergent validity [33, 34].

#### Potential confounders

Potential confounding variables taken into account in our analysis were women’s age, ethnic group, socioeconomic deprivation, highest level of formal education, relationship status and employment. Many of these sociodemographic variables have been previously reported to be linked to social support; for example, experiencing lower levels of social support have been associated with giving birth at an earlier age [12, 35], being from an ethnic minority [36], lower educational attainment [37], lower socioeconomic status [38] and not having or not living with a partner [39]. Index of multiple deprivation (IMD) decile, a common indicator of socioeconomic deprivation in the UK, was obtained by searching participants’ postcodes using a standard online tool [40]. The geographical site where participants were recruited was also included as a potential confounder. Questions and responses relating to sociodemographic information were identical to those from previous Census surveys [41].

We explored the impact of technology use on the association between social support and maternal wellbeing and self-efficacy. Technology use was assessed using the Media and Technology Usage and Attitudes Scale (MTUAS) which has shown high internal consistency, from 0.61 to 0.96 across all 15 subscales [42]. After discussion with the author, to avoid respondent fatigue, nine items were selected from the original scale, which described general aspects of technology use. Items assessed the frequency of text messaging, phone calls, smartphone use, internet searching and social media use. Smartphone use includes browsing the web and using apps of any type on a smartphone or tablet. Internet searching includes searching the web for news and information. General social media use includes checking page, posting photos or commenting on Facebook or other social networks. To make the scale simpler, the number of Likert scale points was reduced from 10 to 6; feedback from pilot respondents had suggested the appropriateness of these changes. Participants indicated the frequency of each of these behaviours, e.g., ‘Check your Facebook page or other social networks’, from 1 (Never/Not applicable) to 6 (Several times a day). Average scores were calculated and ranged from 1 to 6, where 6 corresponded to the highest frequency of technology use.

The MTUAS scale was compared between pregnancy app users and non-pregnancy app users as a test of content validity. The premise underlying this content validity test was that pregnancy app users would show higher frequency of technology use than non-pregnancy app users; this analysis is presented here to increase understanding of the tools used. As hypothesised, participants who reported using pregnancy apps had significantly higher general technology use (mean = 5.12, SD = .03) compared to participants who reported not using pregnancy apps (mean = 4.94, SD = .07), *t* (487) = − 2.84, *p* = 0.005, supporting the content validity of the shorter MTUAS scale used.

### Data analysis

All analysis was completed in Stata 14 [43]. Descriptive statistics were performed to characterise the sample, including median, lower quartile (LQ) and upper quartile (UQ). Categories were re-grouped to remove low number cells and improve the stability of the model for the analysis: ethnicity was divided into two groups 1) White British and 2) Other (other white European, Asian or Asian British, Black or Black British or a Mixed background); education level was split into 1) women with a degree or higher and 2) women without a degree; relationship status was divided into 1) being married or living with partner vs 2) being single, having a partner but not living together or other status; employment comprised of 1) being in paid employment (full-time, part-time, self-employment, or on leave from employment) and 2) not being in paid employment (studying, training, not employed). In the UK, the word ‘degree’ usually refers to an undergraduate academic degree but it is possible that it covers other qualifications in other countries. The variable for the evaluation site was retained without alterations as there was a reasonable number of participants recruited in all of the five sites. Age and IMD decile were used as continuous variables for analysis.

Linear regression models were developed to test the independent associations between each of the potential confounders and social support. The alpha level was set at 0.05. For this analysis we reported the F statistic and *p* value for each of the models overall. In addition, linear regression models were developed in three steps to assess: 1) the unadjusted association between social support and mental well-being, 2) this association adjusting for the potential confounders and, 3) the effect of technology use on the adjusted model. This three-step process was followed for both outcome variables, maternal well-being and self-efficacy. For this analysis, we reported the adjusted R-squared and significance level (alpha = 0.05) for all the models, as well as the regression coefficients and 95% confidence intervals independently for social support (models 1, 2 and 3) and for technology use (model 3).

There were only a small amount of missing data, therefore no imputation procedures were undertaken. Overall scores consisting of sums excluded participants with one or more items missing.

## Results

A total of 492 participants returned a completed baseline questionnaire across all five maternity sites. Baseline characteristics of the study participants are presented in Table 1. On average (median), participants were 28 years old (LQ 24 – UQ 32; overall range 16 to 46), 13 weeks pregnant upon recruitment (LQ 12.4 – UQ 14.4; overall range 7 to 24) and were on the 4th IMD decile (LQ 2- UQ 6; overall range 1 (highest deprivation) to 10 (lowest deprivation)). Approximately half of them had a degree or higher degree (49.3%); of the other half, 18.9% had professional qualifications. The vast majority were married or living with a partner (83.6%) and were in paid employment (86.2%). Most women identified themselves as white British (64.6%); the remaining were other white European (16.3%), Asian or Asian British (13.8%), black or black British (4.0%), mixed (0.6%) or other (0.6%).

**Table 1:** Sample characteristics (*N* = 492)

	n missing (%)	n (%)	Median (LQ - UQ)	Min	Max
Age (years)	19 (3.9%)		28 (24–32)	16	46
Gestation (weeks)	10 (2.0%)		13 (12.4–14.4)	7.7	24.9
IMD decile	15 (3.0%)		4 (2–6)	1	10
Geographical area	0 (0.0%)				
Site 1 (West Midlands)		171 (34.8%)			
Site 2 (London)		140 (28.5%)			
Site 3 (Lancashire)		61 (12.4%)			
Site 4 (East Midlands)		53 (10.8%)			
Site 5 (West Yorkshire)		67 (13.6%)			
Ethnicity	20 (4.1%)				
White British		305 (64.6%)			
Other		167 (35.4%)			
Education Level	11 (2.2%)				
Degree or higher		237 (49.3%)			
No degree		244 (50.7%)			
Relationship status	4 (0.8%)				
Married, or living with partner		408 (83.6%)			
Not married, or not living with partner		80 (16.4%)			
Employment	14 (2.8%)				
In paid employment		412 (86.2%)			
Not in paid employment		66 (13.8%)			

Age data approximated a normal distribution; data for weeks’ gestation and IMD were highly skewed. Therefore, median and LQ-UQ are reported for all of the three variables

Ethnicity: ‘Other’ includes other white, Asian or Asian British, black or black British, mixed, and other

Education level: ‘No degree’ includes those who left school before completing GCSE’s, completed GCSE’s, hold A Levels or an apprenticeship, or professional qualifications

Relationship status: ‘Not married, or not living with partner’ includes single and other (e.g. widow)

Employment: ‘In paid employment’ includes full- and part-time employment, self-employed and on leave from employment; ‘Not in paid employment’ includes studying/training and not in paid employment

*LQ* lower quartile, *UQ* upper quartile; n (%) - denominator is n of participants with data

*IMD* Index of multiple deprivation, ranging from decile 1 (most deprived) to 10 (least deprived)

Data on the exposure variable (perceived social support), outcome variables and technology use, are presented in Table 2. Analysis excluded participants with all of the MTUAS items missing (*n* = 1; 0.2%) or with one or more items missing on the WEMWBS (*n* = 17; 3.5%), antenatal TOPSE (*n* = 44; 8.9%) or MSPSS (*n* = 8; 1.6%). For all the scales used in this study, higher scores meant a higher level of the measured variable and no threshold has been established for ‘high scores’ and ‘low scores’.

**Table 2:** Descriptive data on exposure variable, outcomes and technology use

	Scale score range	n responses missing (*N* = 492)	Median (LQ - UQ)
MSPSS overall	12–84		81.0 (74.0–84.0)
MSPSS Significant other	4–28		28.0 (28.0–28.0)
MSPSS Family	4–28	8 (1.6%)	28.0 (25.0–28.0)
MSPSS Friends	4–28		27.0 (24.0–28.0)
WEMWBS overall	14–70	17 (3.5%)	54.0 (48.0–60.0)
Antenatal TOPSE overall	0–360	44 (8.9%)	319.0 (295.5–340)
MTUAS overall	1–6		5.1 (4.7–5.4)
MTUAS Text messaging	1–6		6.0 (6.0–6.0)
MTUAS Phone calling	1–6	1 (0.2%)	6.0 (5.0–6.0)
MTUAS Smartphone use	1–6		6.0 (6.0–6.0)
MTUAS Internet searching	1–6		6.0 (5.0–6.0)
MTUAS General social media use	1–6		4.0 (3.3–5.0)
		n responses missing (*N* = 492)	n (%)
Uses mobile phone		1 (0.2%)	490 (99.8%)
Uses a tablet (e.g. iPad/Android)		4 (0.8%)	317 (65.0%)
Accesses the internet on phone/tablet		7 (1.4%)	485 (99.8%)
Accesses the internet at home		3 (0.6%)	481 (98.4%)
Uses pregnancy app(s)		3 (0.6%)	356 (72.8%)

MSPSS, WEMWBS and Antenatal TOPSE consist of sum scores. MTUAS consists of mean scores

All four scale variables were not normally distributed, therefore median and LQ-UQ are reported. However, error terms of the outcome variables were normally distributed

*MSPSS* Multidimensional Scale of Perceived Social Support, *MTUAS* Media and Technology Usage and Attitudes Scale, *WEMWBS* Warwick-Edinburgh Mental Wellbeing Scale, *TOPSE* Tool to Measure Parenting Self-Efficacy

The median overall social support (MSPSS) score was 81.0 (IQR 10.0), with little variation between subscales. Median overall scores were 54.0 (IQR = 12.0) for the WEMWBS, and 319.0 (IQR = 44.5) for the antenatal TOPSE. In the MTUAS scale, the average overall score was 5.1 (IQR 0.8), which suggests that technologies were used at least once a day (i.e. on the Likert scale, 5 corresponded to ‘Once a day’). Text messaging, phone calls, smartphone use and internet searching had a median score of 6.0 (IQR varied between 0.0 and 1.0), i.e. ‘Several times a day’. Less frequently, participants used social media - median 4.0 (IQR 1.7) - i.e. ‘Several times a week’. Nearly all participants reported using a mobile phone (*n* = 490, 99.8%), accessed the internet on a mobile phone or tablet (*n* = 485, 99.8%) and accessed the internet at home (*n* = 481, 98.4%). Almost two thirds of women used a tablet (*n* = 317, 65.0%).

Potential confounders for inclusion in the final analysis, including technology use, were pre-specified based on existing literature. Their association with social support was tested through linear regression models (Table 3) prior to the main regression analysis. Social support was significantly associated with IMD decile, F (1, 470) =4.10, *p* = .044, relationship status, F (1, 483) = 17.09, *p* < .001 and with employment status, F (1, 473) = 6.17, *p* = .013. An increase of one decile of IMD decile was associated with a 0.45 increase in social support; not being married or not living with a partner was associated with a 5.51 decrease in social support; not being in paid employment was associated with a 3.64 decrease in social support. None of the other variables revealed significant associations with social support.

**Table 3:** Associations between potential confounders and social support

Potential confounders	F (df)	*p* value
Age (*n* = 468)	F (1) = 0.21	.650
IMD decile (*n* = 470)	F (1) = 5.14	.024*
Ethnicity (*n* = 467)	F(1) = 0.06	.809
Education (*n* = 476)	F(1) = 0.91	.341
Relationship status (*n* = 483)	F(1) = 17.09	<.001**
Employment (*n* = 473)	F(1) = 6.17	.013*
Recruitment site (*n* = 484)	F(4) = 1.58	.178
Technology use (*n* = 483)	F(1) = 0.21	.648

F statistic is reported rather than the coefficient, due to the difficulty in interpreting the coefficients associated to the variable recruitment site which was the only true categorical variable

*n* number of observations; *df* Degrees of Freedom

*p < .05; **p < .001

Further linear regression models were developed to examine the association between social support and each of the outcomes and to what extent technology use affected those associations. All the regression models showed that social support was significantly associated with mental well-being (Table 4) and antenatal self-efficacy (Table 5) with and without adjustment for confounding factors (in all models *p* < .001). When potential confounders were taken into account in model 2, 13% of the variance in mental wellbeing was explained by the model. A 1 point increase on the social support scale was associated with a 0.30 point increase in mental well-being (95% CI 0.22 to 0.38) (Table 4). For antenatal self-efficacy, the model 2, 14% of the variance in antenatal self-efficacy was explained by the model. A 1 point increase in the social support scale was associated with a 1.04 increase in the antenatal self-efficacy scale (95% CI 0.70 to 1.39) (Table 5).

**Table 4:** Associations between mental well-being and both social support and technology use

Model	*R*^2^ adjusted	Social support		Technology use	
		Regression coefficient (SE)	95% CI	Regression coefficient (SE)	95% CI
Model 1 (*n* = 470)	0.12***	0.31 (0.04) ***	0.24 to 0.39		
Model 2 (*n* = 411)	0.13***	0.30 (0.04) ***	0.22 to 0.38		
Model 3 (*n* = 410)	0.13***	0.30 (0.04) ***	0.21 to 0.38	0.39 (0.69)	- 0.97 to 1.75

Model 1: Mental well-being (outcome) and social support (exposure), unadjusted

Model 2: Same as Model 1 adjusting for confounders (age, ethnicity, education, relationship status, employment status, IMD decile and site)

Model 3: Same as Model 2 adjusting for technology use

***p < .001

**Table 5:** Associations between self-efficacy and both social support and technology use

Model	*R*^2^ adjusted	Social support		Technology use	
		Regression coefficient (SE)	95% CI	Regression coefficient (SE)	95% CI
Model 1 (*n* = 446)	0.05***	0.76 (0.16) ***	0.45 to 1.06		
Model 2 (*n* = 400)	0.14***	1.04 (0.18) ***	0.70 to 1.39		
Model 3 (*n* = 399)	0.13***	1.03 (0.18) ***	0.68 to 1.38	0.92 (2.86)	-4.71 to 6.54

Model 1: Self-efficacy (outcome) and social support (exposure), unadjusted

Model 2: Same as Model 1 adjusting for confounders (age, ethnicity, education level, relationship status, employment status, IMD decile and site)

Model 3: Same as Model 2 adjusting for technology use

***p < .001

Technology use was not significantly associated with mental well-being (0.39, 95% CI -0.97 to 1.75) and had no impact on the adjusted model’s goodness of fit (model 3, Table 4). In addition, there was no significant association between technology use and self-efficacy (0.92, 95% CI -4.71 to 6.54) and this variable decreased the model’s goodness of fit by approximately 1% (model 3, Table 5).

## Discussion

To the best of our knowledge, this is the first study to assess the impact of technology use on the association between social support and mental well-being amongst women in early pregnancy. Although the data clearly showed a positive association between social support and maternal well-being, our findings suggest that technology use had no impact on this association. Similarly, social support was positively associated with self-efficacy, which did not change significantly when technology use was taken into account.

Our findings add to the growing body of evidence for the positive association between social support and maternal mental health [9, 10], which has implications for early intervention. Social support may increase well-being both directly, when a person feels integrated in a large social network and indirectly, by acting as a buffer against potentially adverse effects of stressful events [44]. Our study, which used a measure of perceived social support in ‘situations of need’ or ‘when things go wrong’, provides empirical evidence for the buffering role of the support supplied by partner, family and friends. Consistent with this interpretation is the fact that social support was significantly associated with having a partner and with being employed (presumably through interactions in the work place).

The negative association between socioeconomic deprivation and social support found in this study is in line with previous findings [38]. Others have also reported a significant association between social support and maternal age (negative, though not linear) [45], ethnic minority background (negative) [36] and education level (positive) [37] which we failed to observe. The characteristics of our sample may account for these differences. The proportion of participants with a degree or higher degree (49.3%) outnumbered that reported in a previous study [46] and the national average of 42% [47]. Deprivation scores were above what would be expected in the areas where the study was carried out [40]; the mean IMD decile across the five sites (i.e. Local Authority Districts) was 3.2, compared to a mean of 4.3 (SD 2.6) among study participants (higher IMD deciles indicate lower socioeconomic deprivation). This suggested a relatively higher mean socioeconomic status in our sample when compared to the population in those geographical areas from which the participants were recruited. This aspect, coupled with the sample being predominantly white British (reflective of the English population), may explain why levels of social support were above those reported previously [48, 49]: participants in these two studies were from black and minority ethnic groups, a factor that has been found to be linked to lower levels of social support [36].

The unemployment rate (13.8%) was above the national average for females (3.3%) [50]. A post-hoc comparison of mean IMD decile between employed (mean = 4.6, SD = 0.13) and non-employed women (mean = 2.77, SD = 0.24) shows that the latter experience higher levels of deprivation, rather than not working due to lack of need. This suggests that both the most affluent and, to a lesser extent, the least affluent were common in our study which could be related to a number of reasons. For analytical purposes, we grouped participants who reported not being in paid employment (10.5%) with those who were studying or in training (3.3%) resulting in a total of 13.8%. It is possible that some of those studying/in training had a source of income (e.g. postgraduate students on a scholarship). Self-selection, in that more highly educated women are more likely to agree taking part in research, could also have occurred [51]. At the same time, the fact that our study took place in some of the most deprived areas of the country could have led to the enrolment of a considerable number of women who were unemployed.

Measures used in this study have been widely used in antenatal research and allow comparisons between groups, or within groups across time points (no threshold of clinical significance exists to distinguish groups, e.g. high vs low wellbeing). Overall WEMWBS scores in our sample (median 54.0 (12.0)) were slightly above those from another study with prenatal women in the UK (mean 50.1 (7.9)) [52]. Besides chance, a possible explanation for this difference is the gestational stage of participants. In the latter study, women were in the third trimester of pregnancy whereas in ours participants were in their first trimester. The risk of poor mental health (i.e., lower wellbeing) has been found to increase with the greater demands of advancing pregnancy [53]. With regards to the antenatal TOPSE, comparisons are difficult because only postnatal TOPSE data have been published to date. Furthermore, previous assessments of antenatal self-efficacy involved instruments other than the TOPSE and focused on self-efficacy with respect to specific behaviours or tasks, such as breastfeeding [54] or childbirth [55].

Consistent with existing data [56–58], the use of technologies was widespread among participants, reiterating the presence of technology in modern society. However, an important finding of our study is that the associations between social support and both wellbeing and self-efficacy were not significantly affected by the level of technology use. This is somewhat unexpected as there is extensive use of self-care technologies in countries such as the UK or the US, including for the delivery of antenatal and postnatal services [59]. Agencies such as the National Institute for Health and Care Excellence (UK), in their guidance for the management of antenatal and postnatal mental health, include the use of technological devices namely phones and computers [1]. However, there seems to be a paucity of evidence about the impact of technologies on maternal well-being. In keeping with research pointing to the potential negative effects of social media on young people’s mental health [60], postnatal women (*n* = 721, mean age 30.4 years) who made social comparisons on social networking websites were found to be at increased risk of various detrimental outcomes, including perceptions of lower parental competence, lower social support and higher levels of depression [61]. On the other hand, another study with new mothers (*n* = 157, average age 27 years) reported that blogging, but not social networking, was associated with feelings of connection to extended family and friends which then predicted perceptions of social support and, in turn, maternal well-being [21]. According to the authors, these results could be related to the nature of activities involved in blogging and social networking: women were able to share successful parenting experiences on blogs, receive feedback and learn from others while reading blogs, whereas in social networks they could see what friends and family were doing but may not have received much support in return [21]. In fact, learning through others (vicarious experience) is known to enhance self-efficacy [62].

Altogether, these findings suggest that the impact of technology use on antenatal wellbeing is dependent on the type of activity. Our scale of general technology use, derived from a validated tool, focused on more general activities. It is also conceivable that some technologies and online activities yield greater benefits later in pregnancy or postnatally when demands increase. This question warrants further investigation.

Our study is one of the few quantitative studies to date investigating how technology use affects the wellbeing of women in the antenatal period. This topic is highly relevant if we consider the role increasingly played by technologies in the delivery of healthcare services, antenatal or otherwise. However, our study also had a number of limitations. The cross-sectional design of the study can only show if an association exists between variables, not a causal relationship. Degree holders were overrepresented in the sample which limits the generalisability of our findings. The conclusion that technology use has no impact on social support and well-being and self-efficacy requires caution because of the very high levels of general technology use observed in our sample. Ceiling effects have been noted in previous research that evaluated the impact of a school programme to increase technology use and skill, where baseline levels of some activities were already too high to allow room for improvement [63]. This may have happened in our case; a real lack of low technology users may have affected the power of the regression analysis. Although we provide some data supporting the validity of the adapted MTUAS scale, the changes made could also have altered the precision of the original scale.

## Conclusion

Our findings indicate that the use of technologies, in its general form, has no or minimal influence on the association between social and mental well-being and self-efficacy during the early antenatal period. Future research, both quantitative and qualitative, should explore what aspects of technology use can facilitate social networks. Additional work is needed to investigate whether pregnancy-specific and technology-mediated activities, such as sharing pregnancy experiences online or using pregnancy apps, offer more potential to improve the lives of expectant mothers. This should include the development of health literacy skills where needed, such as information-seeking skills and internet access, which can impact positively on the psychological outcomes of perinatal women [64]. The next stage of this project is to evaluate and explore the impact of a pregnancy and parenthood-related technology on antenatal and early postnatal wellbeing and self-efficacy. Understanding the impact of such technologies, if any, is important if we are to develop more effective and cost-effective interventions to support and improve the lives of antenatal and postnatal women and, in turn, that of their children.

## Additional file

Additional file 1: Baseline Questionnaire. (DOCX 532 kb)
