# Supplementary material for: Associations between social support, mental wellbeing, self-efficacy and technology use in first-time antenatal women: data from the BaBBLeS cohort study
Source: BMC Pregnancy Childbirth. 2018 Nov 12;18:441. doi: 10.1186/s12884-018-2049-x (PMC6233574; doi:10.1186/s12884-018-2049-x)
Supplement: Supplementary file 1 — Baseline Questionnaire. (DOCX 532 kb) [file 12884_2018_2049_MOESM1_ESM.docx]

**First questionnaire for the BaBBLeS study**

*Optionally, this questionnaire can be completed online via the link https://www.tinyurl.com/babbles1*

*When prompted, please enter your unique code shown on the top right corner of this page.*

We would be really grateful if you could complete this questionnaire about you as a future parent, your use of technology, your support networks, confidence and well-being.

- Others have found that it takes about 15 minutes to complete.
- If you make a mistake, just cross out the wrong answer and give a new answer.
- Everything you tell us will be treated as confidential.
- Please return the questionnaire in the envelope provided. You can choose to receive a £5 voucher when you send back the completed questionnaire.
- If you would like help to complete the questionnaire please contact: …

Your answers are really important to us. They will help us understand the needs of mothers like you and help develop better ways to support them.

We will contact you again nearer to the birth of your baby with a shorter questionnaire.

**Section 1 – About your pregnancy**

| Y  Y  Y  Y  M  M  D  D  1. When is your baby due?  *Please write in day (DD), month (MM) and year (YYYY)* |
| --- |

2. How do you think you will feed your baby: *Please tick one box only for each row*

|  | Breast feed only | Formula milk only | Both breast feed and formula milk | Not sure |
| --- | --- | --- | --- | --- |
| a. in the first week |  |  |  |  |
| b. by the end of the first month |  |  |  |  |
| c. by the end of three months |  |  |  |  |
| d. at six months |  |  |  |  |

**Section 2: Your use of technology and how you look for information**

| *Please tick one box only on each row, YES or NO* | YES | NO |
| --- | --- | --- |
| 3. Do you use a mobile phone? ………………………………..…………. |  |  |
| 4. Do you use a tablet (e.g. iPad/ Android)? …………………………….. |  |  |
| 5. Do you access the internet on your mobile phone or on your tablet? |  |  |
| 6. Do you access the internet at home? ………………............................. |  |  |

| 7*. The next question is about your use of mobile phones, tablets, laptops or any other device. Please indicate how often you do each of the following activities, by ticking one box for each row.^1^* | | | | | | |
| --- | --- | --- | --- | --- | --- | --- |
|  | Never/ Not applicable | Less than once a week | Once a week | Several times a week | Once a day | Several times a day |
| a. Send and receive text messages on a mobile phone or tablet |  |  |  |  |  |  |
| b. Make and receive calls on a mobile phone or tablet |  |  |  |  |  |  |
| c. Browse the web on a mobile phone or tablet |  |  |  |  |  |  |
| d. Use apps (for any purpose) on a mobile phone or tablet |  |  |  |  |  |  |
| e. Search the internet for news on any device |  |  |  |  |  |  |
| f. Search the internet for information on any device |  |  |  |  |  |  |
| g. Check your Facebook page or other social networks on any device |  |  |  |  |  |  |
| h. Post photos on Facebook or other social networks |  |  |  |  |  |  |
| i. Comment on postings, status updates, photos, etc. on Facebook or other social networks. |  |  |  |  |  |  |

| 8. Did you or do you use any app specifically about pregnancy (on a phone, tablet or computer)? *Please tick one box only, YES or NO* | |
| --- | --- |
| Yes *Please name*:  a) …………………………………………..  b) …………………………………………..  c) ………………………………………….. | No  *If you have answered No, please go to question 10* |

| 9. How did you hear about the pregnancy app(s) you are using or used? *Please tick all that apply* |
| --- |
| a. Midwife…………………………...………………………………...…………… |
| b. Health Visitor……………............................................................................ |
| c. GP…………………...………………………………………..…………………. |
| d. Other health professional(s).......................................................................  *Please say which one(s)*: _________________________________ |
| e. Partner…………………………………………………………………….……..  f. Friends, other mothers-to-be or new mothers……...................................... |
| g. Posters at GP surgery, clinic or hospital…………...................................... |
| h. Through other apps that I have used before……….…...………….….........  *Please say which one(s)*: _________________________________ |
| i. Internet search………......................................................................………. |
| j. Books or magazines……………………………………………....................... |
| k. Other. *Please specify*: ___________________________________ |
|  |
| 10. Where do you look or who do you ask for information about pregnancy and parenthood? *Please tick all that apply* |
| a. Midwife…………………………………………………………………….……... |
| b. Health Visitor…………………………………………………………………….. |
| c. GP…………………………………………………………………………........... |
| d. Other health professional(s)……………………………………………........... |
| *Please say which one(s)*: ________________________________ |
| e. Partner…………………………………………………………………………… |
| f. Friends, other mothers-to-be or new mothers….…………………………..… |
| g. Posters at GP surgery, clinic or hospital…………………………………...… |
| h. Apps……………………………………………………………………………… |
| i. Internet search……………………………………………………………….…. |
| j. Books or magazines……………………………………………………........... |
| k. Other. *Please specify*: _____________________________________ |

**Section 3 – About how you might feel when your baby is born**

11*. We would like to know how you are feeling now, while you are pregnant, about becoming a mother. We understand that it might be difficult for you to imagine how you will interact with you baby, but we would be grateful if you can complete each of the questions as best as you can.*

*We would also like to know about the support you receive from other people, and how you feel emotionally.*

| *- The following section is about emotion and affection -*  Using the scale below^2^, **please enter in the boxes how much you agree with each statement**. The scale ranges from 0 (completely disagree) to 10 (completely agree). You may use any number between 0 and 10. Please answer all statements.  0 1 2 3 4 5 6 7 8 9 10  Completely disagree Moderately agree Completely agree |
| --- |

1. I will be able to show affection towards my baby…………………………………………………………...…….

2. I will be able to recognise when my baby is happy or sad…………………………………............................

3. I am confident my baby will be able to come to me if he/she is unhappy…………...……………………….

4. When my baby is sad I will understand why……………………………………..………………………………

5. I will have a good relationship with my baby………………………………...………………...........................

6. I will find it hard to cuddle my baby……………………………………………………………...........................

| *- The following section is about play and enjoyment -*  Using the scale below^2^, **please enter in the boxes how much you agree with each statement**. The scale ranges from 0 (completely disagree) to 10 (completely agree). You may use any number between 0 and 10. Please answer all statements.  0 1 2 3 4 5 6 7 8 9 10  Completely disagree Moderately agree Completely agree |
| --- |

7. I will be able to have fun with my baby……………………………………………………………………….…..

8. I will be able to enjoy each stage of my baby’s development…………………………………………….…...

9. I will be able to have nice days with my baby………………………………………..…………………..……..

10. I will be able to plan activities that my baby will enjoy………………………………………….………..……

11. Playing with my baby will come easily to me………………………………….……………..........................

12. I will be able to help my baby reach their full potential………………………..………………………………

| *- The following section is about empathy and understanding -*  Using the scale below^2^, **please enter in the boxes how much you agree with each statement**. The scale ranges from 0 (completely disagree) to 10 (completely agree). You may use any number between 0 and 10. Please answer all statements.  0 1 2 3 4 5 6 7 8 9 10  Completely disagree Moderately agree Completely agree |
| --- |

13. I will be able to explain things patiently to my baby………………………………………………………….

14. I will be able to get my baby to listen to me…………………………………………………...……….……..

15. I will be able to comfort my baby…………………………………………...………….………......................

16. I will be able to listen to my baby………………………………………………...…….………………………

17. I will be able to put myself in my baby’s shoes……………………………………….………………………

18. I will understand my baby’s needs……………………………………………………..……………………...

| *- The following section is about pressures -*  Using the scale below^2^, **please enter in the boxes how much you agree with each statement**. The scale ranges from 0 (completely disagree) to 10 (completely agree). You may use any number between 0 and 10. Please answer all statements.  0 1 2 3 4 5 6 7 8 9 10  Completely disagree Moderately agree Completely agree |
| --- |

19. It will be difficult to cope with other people’s expectations of me as a parent…………………………......

20. I will not be able to assert myself when other people will tell me what to do with my baby…………..….

21. Listening to other people’s advice will make it hard for me to decide what to do……………………..….

22. I will be able to say ‘no’ to other people if I don’t agree with them……………………………………..…..

23. I will be able to ignore pressure from other people to do things their way………………………………..

24. I will not feel a need to compare myself to other parents……………………………………………………

| *- The following section is about self-acceptance -*  Using the scale below^2^, **please enter in the boxes how much you agree with each statement**. The scale ranges from 0 (completely disagree) to 10 (completely agree). You may use any number between 0 and 10. Please answer all statements.  0 1 2 3 4 5 6 7 8 9 10  Completely disagree Moderately agree Completely agree |
| --- |

25. I know I will be a good enough parent………………………………………………………………….………

26. I will manage the pressures of parenting as well as other parents do……………………………….…….

27. I will not do that well as a parent…………………………………………..……………………………….….

28. As a parent I will be able to take most things in my stride…………………………………………….…….

29. I will be able to be strong for my baby…………………………………………………..…………….………

30. My baby will feel safe around me………………………………………………………..…………….………

| *- The following section is about learning and knowledge -*  Using the scale below^2^, **please enter in the boxes how much you agree with each statement**. The scale ranges from 0 (completely disagree) to 10 (completely agree). You may use any number between 0 and 10. Please answer all statements.  0 1 2 3 4 5 6 7 8 9 10  Completely disagree Moderately agree Completely agree |
| --- |

31. I will be able to recognise developmental changes in my baby………………………………….………...

32. I will be able to share ideas with other parents……………………………………………..........................

33. I will be able to learn and use new ways of dealing with my baby…………………………………………

34. I will be able to make the changes needed to improve my baby’s behaviour…………………………….

35. I will be able to overcome most problems with a bit of advice…………………………….........................

36. Knowing that other people have similar difficulties with their babies will make it easier for me…………

12. *About the support you receive from other people* - *We are interested in how you feel about the following statements^3^. Read each statement carefully. Indicate how you feel about each statement by ticking one box only for each row.*

|  | Very Strongly Agree | Strongly Agree | Mildly Agree | Neutral | Mildly Disagree | Strongly Disagree | Very Strongly Disagree |
| --- | --- | --- | --- | --- | --- | --- | --- |
| a. There is a special person who is around when I am in need. |  |  |  |  |  |  |  |
| b. There is a special person with whom I can share my joys and sorrows. |  |  |  |  |  |  |  |
| c. My family really tries to help me. |  |  |  |  |  |  |  |
| d. I get the emotional help and support I need from my family. |  |  |  |  |  |  |  |
| e. I have a special person who is a real source of comfort to me. |  |  |  |  |  |  |  |
| f. My friends really try to help me. |  |  |  |  |  |  |  |
| g. I can count on my friends when things go wrong. |  |  |  |  |  |  |  |
| h. I can talk about my problems with my family. |  |  |  |  |  |  |  |
| i. I have friends with whom I can share my joys and sorrows. |  |  |  |  |  |  |  |
| j. There is a special person in my life who cares about my feelings. |  |  |  |  |  |  |  |
| k. My family is willing to help me make decisions. |  |  |  |  |  |  |  |
| l. I can talk about my problems with my friends. |  |  |  |  |  |  |  |

| 13. *About how you feel emotionally - Below are some statements about feelings and thoughts.^4^ Please* ***circle*** *the number that best describes your experience of each of the statements over the* ***last 2 weeks.*** | | | | | |
| --- | --- | --- | --- | --- | --- |
|  | None of the time | Rarely | Some of the time | Often | All of the time |
| a. I’ve been feeling optimistic about the future | 1 | 2 | 3 | 4 | 5 |
| b. I’ve been feeling useful | 1 | 2 | 3 | 4 | 5 |
| c. I’ve been feeling relaxed | 1 | 2 | 3 | 4 | 5 |
| d. I’ve been feeling interested in other people | 1 | 2 | 3 | 4 | 5 |
| e. I’ve had energy to spare | 1 | 2 | 3 | 4 | 5 |
| f. I’ve been dealing with problems well | 1 | 2 | 3 | 4 | 5 |
| g. I’ve been thinking clearly | 1 | 2 | 3 | 4 | 5 |
| h. I’ve been feeling good about myself | 1 | 2 | 3 | 4 | 5 |
| i. I’ve been feeling close to other people | 1 | 2 | 3 | 4 | 5 |
| j. I’ve been feeling confident | 1 | 2 | 3 | 4 | 5 |
| k. I’ve been able to make up my own mind about things | 1 | 2 | 3 | 4 | 5 |
| l. I’ve been feeling loved | 1 | 2 | 3 | 4 | 5 |
| m. I’ve been interested in new things | 1 | 2 | 3 | 4 | 5 |
| n. I’ve been feeling cheerful | 1 | 2 | 3 | 4 | 5 |

**Section 4 – About your background**

| D  D  M  M  Y  Y  Y  Y  14. What is your date of birth?  *Please write in day (DD), month (MM) and year (YYYY)* |
| --- |

15. What is your ethnic group? *Please tick one box only*

*White*:

a. British b. Irish c. Other White European

d. Other *Please specify:* ……………………………………………….

*Asian or Asian British*:

e. Pakistani f. Bangladeshi g. Indian h. Chinese

i. Other *Please specify:* ……………………………………………….

*Black or Black British*:

j. Caribbean k. African

l. Other *Please specify:* ………………………………………………..

*Mixed background*:

m. White & Black Caribbean n. White & Black African

o. White & Asian

p. Other *Please specify:* ………………………………………………..

*q. Any other ethnic group* *Please specify:* ……………………………………….

*r. I do not wish to say*

16. What is the highest level of education that you reached? *Please tick one box only*

a. Left school before completing GCSEs……………………………..

b. GCSEs………………………………………………………..............

c. A Levels/Scottish Highers or International Baccalaureate………

d. Apprenticeship…………………………………………………….....

e. Professional qualifications………………………………………….

f. First Degree……………………………………………………….....

g. Higher degree or above………………………………………….....

17. What best describes your current status? *Please tick one box only*

a. Married or living with your partner………

b. Single………………………………………

c. Have a partner but not living together.........

d. Other…………………………………............ *Please specify*: ______________________________

18. What best describes your current employment? *Please tick one box only*

a. In paid Full time employment…………………………………………

b. In paid Part time employment………………………………………..

c. Self-employed or freelance…………………………………………..

d. Studying or in training………………………………………………...

e. On Maternity Leave or Sick Leave from full time employment…..

f. On Maternity Leave or Sick Leave from part-time employment…

g. Not in paid employment……………………………………………...

Y

Y

Y

Y

M

M

D

D

19. Date when you finished completing this questionnaire:

*Please write in day (DD), month (MM) and year (YYYY)*

20. Please feel free to leave any comments in the box below relating to any of your answers to this questionnaire or suggestions for improvement:

…………………………………………………………………………………………………………………………………………………………………

………………………………………………………………………………………………………………………………………………………………………………………………………………………………………………………………………………………………………………………………………………………………………………………………………………………………………………………………………………………………………

…………………………………………………………………………………………………………………………………………………………………

21. What are the first three or four digits of your postcode?

**Thank you** very much for filling in this questionnaire.

Please check that you have answered all the questions.

Please send this back to us in the **envelope provided** together with the **consent form** completed and signed by you, and the completed **contact details form**. Don’t forget to tick the box if you would like to receive a £5 voucher!

Our address: BABBLeS,

University of the West of England, Bristol

Centre for Child & Adolescent Health

Oakfield House, Oakfield Grove

Clifton

Bristol BS8 2BN

*This project is a collaboration between:*


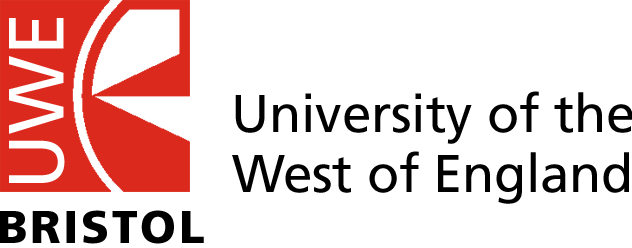

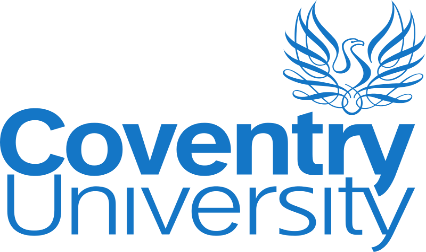


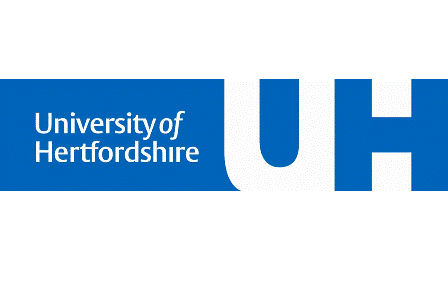


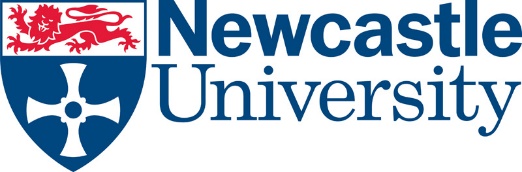


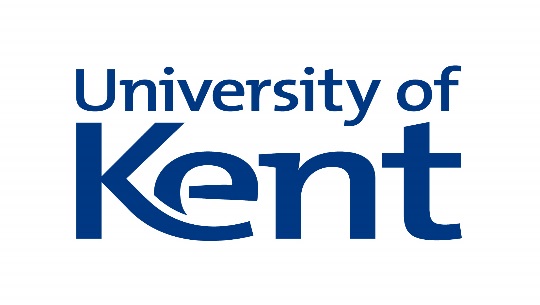


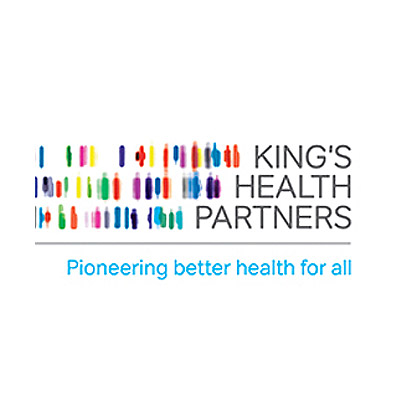


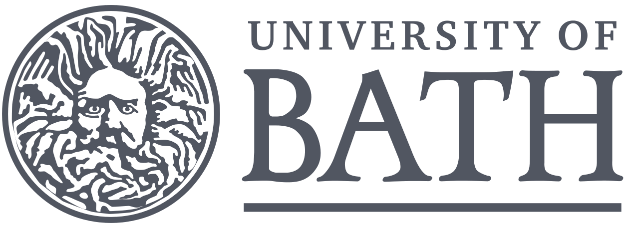


*Sources of questions (superscripts):*

1. *Adapted from the Media and Technology Usage and Attitudes Scale, Rosen et al. 2013*
2. *Adapted from the TOPSE, a tool to measure Parenting Self-Efficacy, Kendall S. & Bloomfield L. 2005, available on* [*www.topse.org.uk*](http://www.topse.org.uk)
3. *Zimet et al. (1988), The multidimensional scale of perceived social support (MSPSS).*
4. *Warwick-Edinburgh Mental Well-being Scale (WEMWBS), NHS Health Scotland, University of Warwick and University of Edinburgh, 2006*
